# An Endoscopic Approach as a Salvage Strategy in Complicated Perforated Gastric Ulcer: A Case Report

**DOI:** 10.7759/cureus.87635

**Published:** 2025-07-09

**Authors:** Juan M Reyes-Morales, Yoeli M Escandon-Espinoza, Katia D Lopez-Garcia, Roberto A Alvarado-Hernández, Victor M Ayuso-Diaz, Raúl J Moguel-Canto

**Affiliations:** 1 Gastrointestinal Endoscopy, Instituto de Seguridad y Servicios Sociales de los Trabajadores del Estado-Hospital Regional de Alta Especialidad Bicentenario de la Independencia, Tultitlan, MEX; 2 Research and Education, Medical Care and Research Center, Yucatan, MEX; 3 Genomics-Metabolics, Marista University of Merida, Yucatan, MEX; 4 Medical Education and Simulation, Universidad Autónoma de Yucatán (UADY), Yucatan, MEX

**Keywords:** damage control surgery, endoscopic salvage therapy, foley catheter drainage, gastric perforation, gastrocutaneous fistula, minimally invasive management, percutaneous endoscopic gastrostomy

## Abstract

A peptic ulcer is a common gastrointestinal condition, the most serious complication of which is perforation. This is often associated with high morbidity and mortality, particularly in patients taking chronic corticosteroids and non-steroidal anti-inflammatory drugs (NSAIDs). Here, we present the case of a 44-year-old Mexican woman with rheumatoid arthritis who presented with acute abdominal pain and clinical signs of peritonitis. Diagnostic imaging confirmed a perforated prepyloric gastric ulcer with pneumoperitoneum. Following multiple unsuccessful surgical interventions due to tissue necrosis and persistent peritonitis, a controlled gastrocutaneous fistula was created using a Foley catheter to control the damage. Following clinical stabilization, persistent leakage prompted an endoscopic approach: the Foley catheter was replaced with a percutaneous endoscopic gastrostomy catheter using the Gauderer-Ponsky technique, two hemoclips were applied to achieve tight closure of the fistulous tract, and a nasojejunal catheter was inserted to restore enteral nutrition. The patient made a favorable clinical recovery, with resolution of the fistula and satisfactory re-establishment of nutrition, and was discharged home. This case illustrates an alternative salvage approach involving endoscopic techniques for damage control and nutritional restoration in patients with complicated gastric ulcer perforations and failed surgical treatment.

## Introduction

A gastric ulcer is a circumscribed loss of tissue affecting the mucosal, submucosal, and muscular layers of the stomach. It occurs in areas that are particularly vulnerable to the action of hydrochloric acid and pepsin [1]. The most serious complication of this pathology is perforation, which has an incidence rate of 1% to 6% and is the initial presentation in one-third of cases [2]. This event is associated with significantly high mortality, particularly among older and male patients. The chronic use of non-steroidal anti-inflammatory drugs (NSAIDs) is considered the most important risk factor in the development of gastric perforation. It is estimated that between 33% and 70% of patients with this complication have a history of using NSAIDs [3]. Late diagnosis can increase the lethality rate to over 90%, compared to under 10% with timely intervention [4].

Standard surgical treatment includes primary closure, an omental patch (the Graham technique), or a combination of both. However, in complex clinical scenarios such as persistent peritonitis, necrotic tissue, or extensive transmural inflammation, these techniques may be unfeasible. In such situations, damage control by establishing a controlled fistula has been proposed as a surgical salvage strategy [5].

The insertion of a balloon Foley catheter at the perforation site is intended to create a controlled gastrocutaneous fistula for the targeted drainage of gastric contents, the reduction of intraluminal pressure, and the minimization of peritoneal contamination [6]. Although this technique is not widely included in guidelines, it has been successfully employed in cases where primary closure is technically impossible or dangerous. In a Mexican study, Echaverry-Navarrete et al. documented the use of this technique in patients with severe gastric erosion due to gastric banding. Spontaneous closure of the tract was achieved after the device and temporary Foley tube were removed [5].

Gachabayov et al. emphasize that the presence of friable tissues, extensive perforations, and generalized sepsis constitutes scenarios in which definitive techniques should be deferred or even avoided, favoring temporary interventions aimed at physiologically stabilizing the patient. In this context, the Foley catheter does not act as a definitive therapeutic device, but rather as a functional bridge that enables the patient to be stabilized and subsequent closure of the fistulous tract to be planned, either spontaneously or directed [6].

Given the favorable clinical evolution and persistent leakage, it has been suggested that this strategy should be supplemented with endoscopic closure techniques, such as percutaneous endoscopic gastrostomy (PEG), hemoclips, and nasojejunal tube placement, in order to re-establish enteral nutrition and encourage the closure of the fistulous tract. Recent literature has documented the success of these minimally invasive techniques, even in cases of refractory postsurgical fistulas [7].

## Case presentation

A 44-year-old female patient with a history of rheumatoid arthritis was receiving chronic treatment with glucocorticoids and NSAIDs. She attended the emergency department with sudden abdominal pain initially located in the epigastrium, which subsequently spread. On admission, the clinical presentation was consistent with overt peritoneal irritation (positive Blumberg's sign, involuntary abdominal rigidity, and generalized hypersensitivity), accompanied by signs of systemic sepsis.

Initial paraclinical studies revealed leukocytosis of 13,000 cells/μL, with 90% neutrophilia, indicative of systemic inflammation (see Table 1). An abdominal radiograph in the standing position showed bilateral subdiaphragmatic pneumoperitoneum, confirming the presumptive diagnosis of an acute abdomen secondary to perforation of the hollow viscera.

**Table 1:** Laboratories at admission

Parameter	Patient's result	Reference range
White blood cells (WBCs)	13,000/μL	4,000-11,000/μL
Neutrophils (%)	90%	40%-75%
Hemoglobin	11.7 g/dL	12-16 g/dL
Hematocrit	35.6%	36%-46%
Glucose	89 mg/dL	70-100 mg/dL
Urea	43 mg/dL	15-40 mg/dL
Blood urea nitrogen (BUN)	20.1 mg/dL	7-20 mg/dL
Sodium (Na⁺)	142 mmol/L	135-145 mmol/L
Potassium (K⁺)	4.5 mmol/L	3.5-5.0 mmol/L
Chloride (Cl⁻)	113 mmol/L	98-107 mmol/L
Prothrombin time (PT)	20.0 s	11-13.5 s
International normalized ratio (INR)	1.26	0.8-1.2
Partial thromboplastin time (PTT)	37.9 s	25-35 s

Upon diagnosis of gastric perforation, the initial surgical intervention consisted of an exploratory laparotomy. This revealed a 1.5 cm prepyloric gastric ulcer with active leakage and fibrinous peritonitis. A two-layer primary closure was attempted using absorbable sutures, reinforced with an omental (Graham) patch. The abdominal cavity was thoroughly irrigated, and bilateral subhepatic and paracolic drains were inserted before temporary abdominal closure.

During the immediate postoperative period, the patient was admitted to the intensive care unit due to hemodynamic instability and the need for invasive mechanical ventilation. Her condition progressively deteriorated, with recurrent intra-abdominal infections necessitating two additional surgical interventions on postoperative days 7 and 10. The second laparotomy revealed purulent peritonitis and dehiscence of the primary gastric closure. Despite extensive peritoneal lavage and drainage, resuturing was not attempted due to the fragility of the surrounding tissue. A third surgical exploration revealed similar findings. Given the progressive inflammation and increased risk of recurrent dehiscence, surgical closure was deferred. Open abdominal management with abdominal packing was maintained.

**Figure 1:**
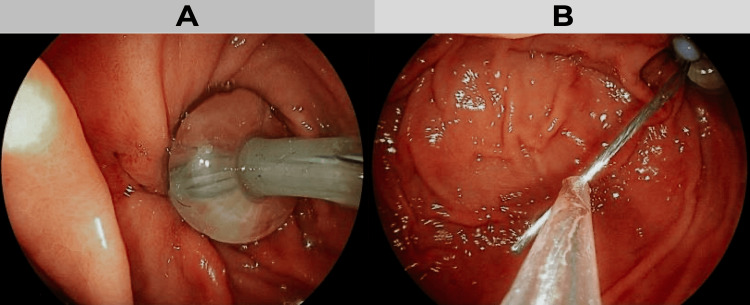
Initial endoscopic management of the gastrocutaneous fistula (A) Visualization of the intragastric Foley tube balloon occupying the prepyloric perforation site. (B) Cannulation of the fistulous tract with a hydrophilic guidewire in preparation for PEG placement in a second-stage procedure. PEG: percutaneous endoscopic gastrostomy

A fourth surgical intervention was performed on postoperative day 14 due to persistent sepsis and a deterioration in abdominal findings. Intraoperative exploration confirmed that the gastric defect was still present and was now surrounded by devitalized tissue, necrotic margins, and severe transmural inflammation. Due to the poor quality of the tissue, it was deemed unfeasible to attempt surgical closure again. Instead, a controlled gastrocutaneous fistula was created by inserting a balloon Foley catheter through the existing fistulous tract into the gastric lumen, thereby achieving external gastric decompression. The peritoneal drains were repositioned, and negative pressure wound therapy was continued as part of the open abdomen management strategy. Figures 1A, 1B illustrate the initial endoscopic management performed after surgical stabilization.

Following hemodynamic stabilization and clinical improvement, enteral feeding was initiated. However, leakage of food and air through the cutaneous fistulous tract was observed, resulting in a referral to the endoscopy service. During the initial postoperative upper endoscopy on day 15, the Foley balloon was visualized within the gastric lumen at the site of the prepyloric perforation, thereby obstructing gastric outflow. The Foley balloon was removed, and the fistulous tract was cannulated with a hydrophilic guidewire under direct vision (Figure 1B). Due to persistent contamination and tissue fragility, the procedure was staged.

**Figure 2:**
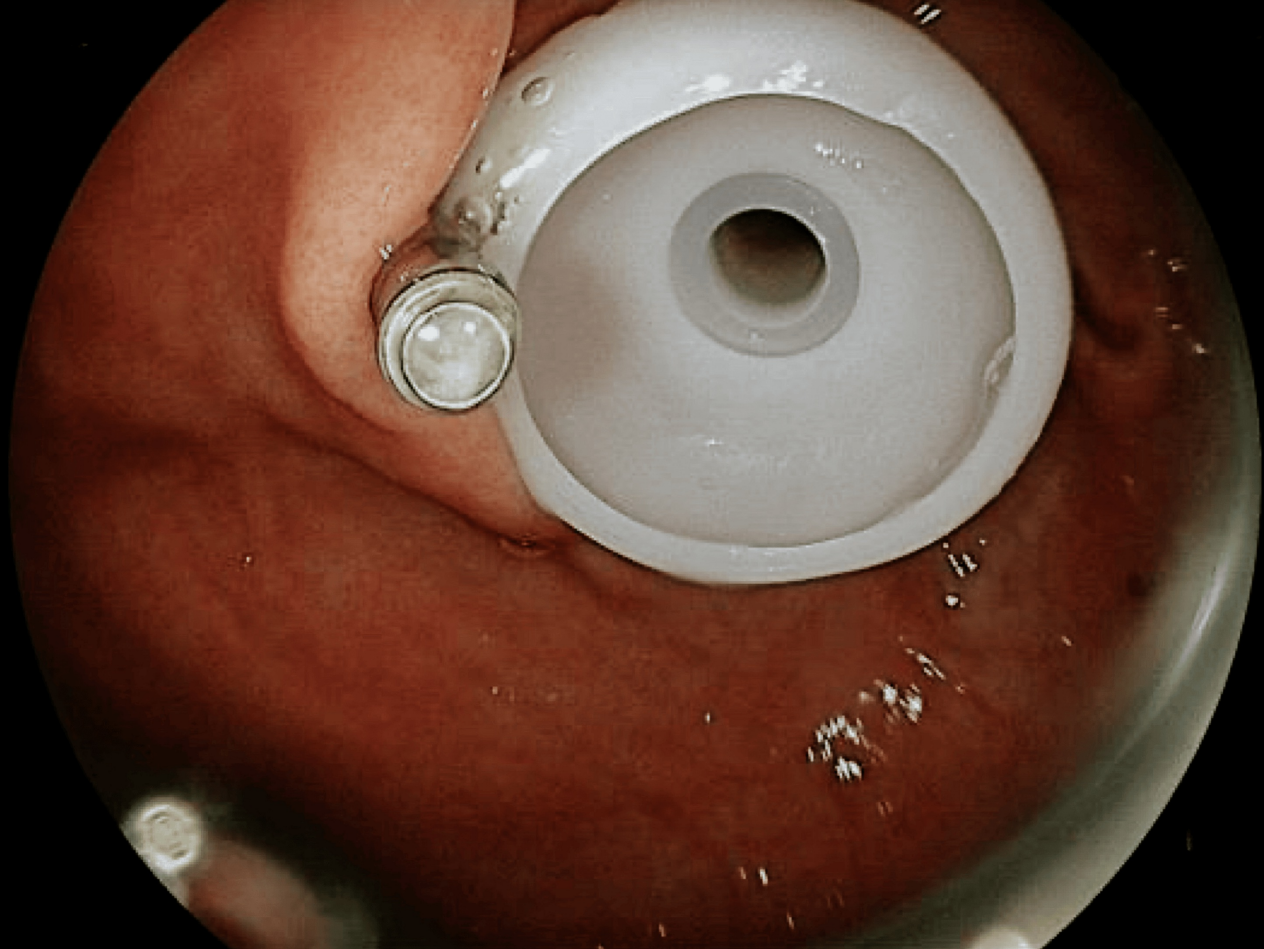
Percutaneous endoscopic gastrostomy (PEG) tube placement was performed using the Gauderer-Ponsky technique A 24 Fr caliber PEG tube was placed using the Gauderer-Ponsky technique. Evidence of correct intragastric tube positioning was obtained, confirming functional access to the gastric cavity.

A second endoscopic session, performed 48 hours later, enabled the placement of a 24 Fr PEG tube using the Gauderer-Ponsky technique (Figure 2). Two hemoclips were placed adjacent to the fistula to minimize leakage around the tube. 

**Figure 3:**
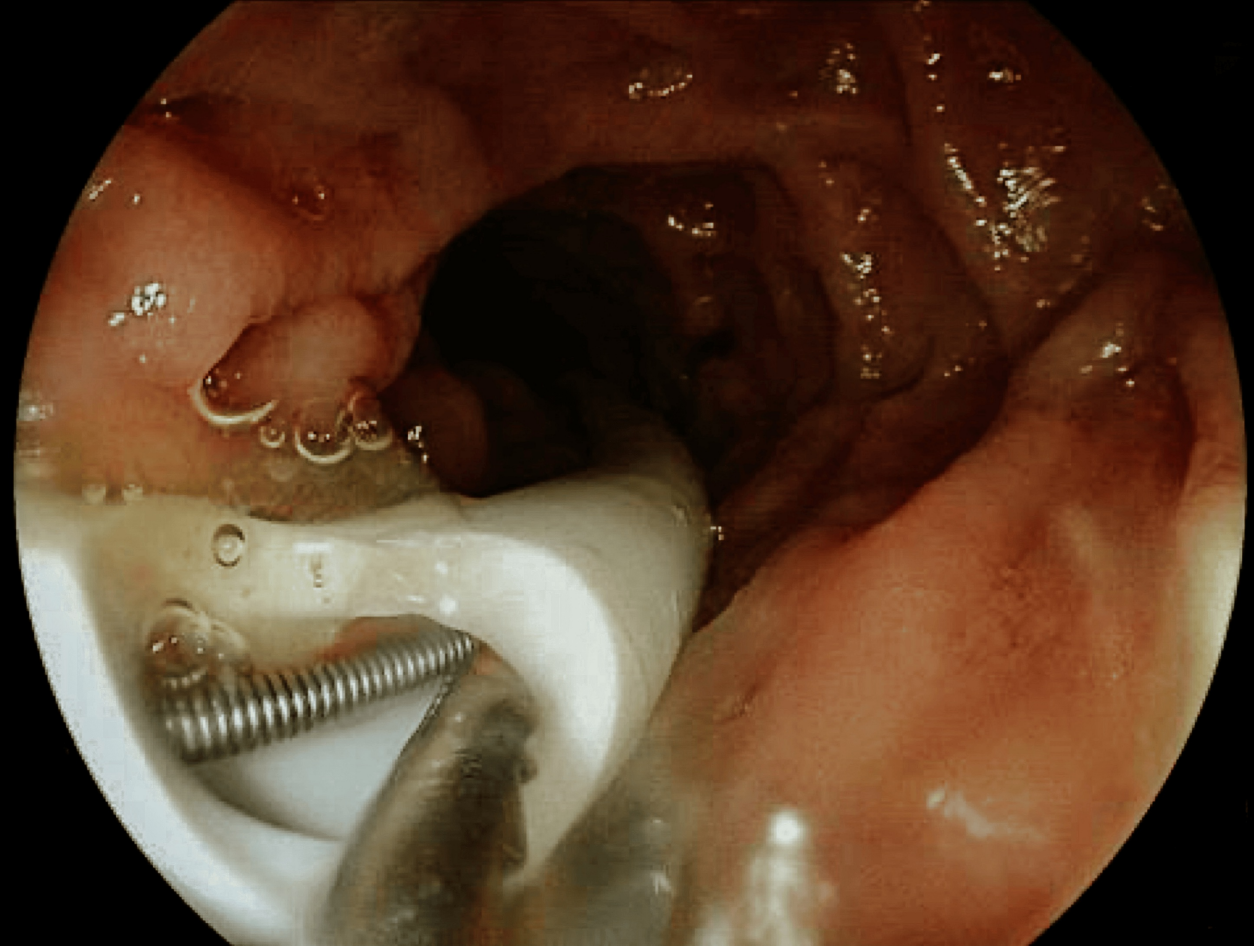
Endoscopic confirmation of PEG integrity and hemostatic clip placement Endoscopic control image showing correct positioning of the PEG tube and application of hemoclips adjacent to the fistulous tract. The absence of leakage confirmed successful closure of the peri-tube pathway and enabled safe resumption of enteral nutrition. PEG: percutaneous endoscopic gastrostomy

During a second endoscopic procedure, a 16 Fr nasojejunal tube was inserted and insufflation was performed to confirm the absence of air leakage, enabling the safe resumption of enteral nutrition (Figure 3). The patient made a favorable recovery, with a progressive decrease in fistulous output, tract healing, and adequate tolerance of the enteral route. They were discharged home two weeks after the procedure and are under ambulatory surgery follow-up.

## Discussion

Peptic ulcer is the most common cause of gastric perforation, with an incidence rate far exceeding that of other less common causes such as trauma, neoplasia, or iatrogenic perforations [1]. Initial management should include fasting, controlling sepsis with antibiotic therapy, and providing drainage via surgical or percutaneous methods. It should also include providing parenteral nutrition, quantifying fistulous output, and accurately identifying the leak site [4,5,8]. Surgical resolution remains the primary treatment in most cases, with either a laparoscopic or open approach being recommended, depending on the patient's hemodynamic status. In complicated cases, such as the one presented here, the open approach is preferred due to greater accessibility and control of the septic focus. Closure can be achieved with a simple suture, an epiploic patch (Graham technique), or a combination of the two. The latter is the most recommended option due to its efficacy in high-risk patients [9,10].

Although not a first-line therapy, endoscopic repair has been shown to be useful in certain situations, particularly for small (less than 2 cm) iatrogenic perforations in stable patients with no signs of peritonitis [9-11]. In contrast, wide or distal perforations require more aggressive techniques, such as partial gastrectomy and reconstruction, whenever the patient's condition permits [12]. If primary closure fails and resection is not feasible due to adverse clinical conditions, damage control involving the creation of a temporary controlled gastrocutaneous fistula may be the only safe option.

Several endoscopic techniques have been used to manage gastric leaks, including hemoclips, over-structure clips (Ovesco Endoscopy AG, Tübingen, Germany), stents, tissue adhesives, and intraluminal negative pressure (vacuum-assisted closure (VAC)) therapy systems [13]. These options require adequate patient selection and experience of the treating team. In this case, hemoclips were chosen because they were readily available, could be deployed easily through a standard gastroscope, and could approximate the margins of the fistulous tract without causing further tissue damage. Their application helped minimize peritubal leakage, stabilize the tract, and improve the effectiveness of PEG tube feeding.

In this case, however, the patient developed a prepyloric perforation complicated by persistent peritonitis and recurrent surgical dehiscence, necessitating multiple reinterventions. Poor tissue quality prevented safe surgical closure, so a controlled gastrocutaneous fistula was formed using a Foley catheter as a damage control measure. Although this technique is unconventional, it has been reported in the literature as an effective method of decompressing the gastric cavity in cases of severe inflammation and fragile tissue. It provides a temporary solution until a definitive solution can be implemented [4-6].

The three open surgical interventions failed due to progressive tissue degradation and ongoing intra-abdominal sepsis. The first laparotomy included a standard two-layer primary closure, which was reinforced with an omental patch. However, purulent peritonitis and dehiscence were found during the second and third surgical re-explorations, and the gastric walls were too friable for resuturing. By the time of the fourth surgery, the tissue had become necrotic and was severely inflamed, making further closure unsafe. These findings justified transitioning to a damage control approach, followed by endoscopic salvage.

Despite controlled drainage, leakage of gastric contents and clinical deterioration persisted, so an endoscopic rescue approach was chosen. A PEG tube was inserted using the Gauderer-Ponsky technique alongside the placement of hemoclips adjacent to the fistulous tract, as well as a nasojejunal tube for enteral nutrition. While this combination is not part of the conventional treatment for gastric leaks, it has produced good results in small-scale studies involving patients with small defects or surgical contraindications [13].

This case highlights the importance of tailoring management strategies for patients with a poor prognosis following gastric perforation, including the consideration of non-conventional salvage techniques. In this case, combining sepsis control, endoscopic closure of the defect, and adequate nutritional support enabled clinical recovery and spontaneous resolution of the fistula tract. We propose that, in selected cases where standard surgical strategies have failed, this approach may represent a safe and effective alternative.

## Conclusions

Gastric perforation is a serious complication associated with high morbidity and mortality rates, particularly among patients with comorbidities or failed surgical repair. In the present case, a favorable clinical outcome was achieved through a multidisciplinary strategy combining initial surgical damage control with subsequent endoscopic salvage therapy. The use of a PEG and a nasojejunal tube allowed restoration of the enteral route and contributed to clinical stabilization.

This experience suggests that minimally invasive approaches may serve as valuable adjuncts in selected high-risk cases where conventional surgical options are limited or have failed. However, the decision to pursue endoscopic or alternative strategies should be based on multiple factors, including tissue viability, patient stability, surgical findings, and the expertise of the treating team.
